# Relationships Between Perivascular Adipose Tissue and Abdominal Aortic Aneurysms

**DOI:** 10.3389/fendo.2021.704845

**Published:** 2021-06-14

**Authors:** Tongtong Ye, Guangdong Zhang, Hangyu Liu, Junfeng Shi, Hongyan Qiu, Yongping Liu, Fang Han, Ningning Hou

**Affiliations:** ^1^ Department of Endocrinology, Affiliated Hospital of Weifang Medical University, Weifang, China; ^2^ Clinical Research Center, Affiliated Hospital of Weifang Medical University, Weifang, China; ^3^ Department of Ophthalmology, Weifang Eye Hospital, Weifang, China; ^4^ Department of Pathology, Affiliated Hospital of Weifang Medical University, Weifang, China

**Keywords:** abdominal aortic aneurysm, perivascular adipose tissue, obesity, vascular, vascular diseases

## Abstract

Abdominal aortic aneurysms (AAAs) are typically asymptomatic, and there is a high mortality rate associated with aneurysm rupture. AAA pathogenesis involves extracellular matrix degradation, vascular smooth muscle cell phenotype switching, inflammation, and oxidative stress. There is increasing evidence of excessive adipocyte accumulation in ruptured AAA walls. These excessive numbers of adipocytes in the vascular wall have been closely linked with AAA progression. Perivascular adipose tissue (PVAT), a unique type of adipose tissue, can be involved in adipocyte accumulation in the AAA wall. PVAT produces various chemokines and adipocytokines around vessels to maintain vascular homeostasis through paracrine and autocrine mechanisms in normal physiological conditions. Nevertheless, PVAT loses its normal function and promotes the progression of vascular diseases in pathological conditions. There is evidence of significantly reduced AAA diameter in vessel walls of removed PVAT. There is a need to highlight the critical roles of cytokines, cells, and microRNA derived from PVAT in the regulation of AAA development. PVAT may constitute an important therapeutic target for the prevention and treatment of AAAs. In this review, we discuss the relationship between PVAT and AAA development; we also highlight the potential for PVAT-derived factors to serve as a therapeutic target in the treatment of AAAs.

## Introduction

Aortic aneurysms are irreversible, permanent manifestations of local vasodilation that can be either thoracic or abdominal; most comprise abdominal aortic aneurysms (AAAs) (1). AAAs are pathological dilations that are 1.5-fold larger than the normal aortic diameter. There is increasing evidence of excessive adipocyte accumulation in ruptured AAA walls (2). These excessive numbers of adipocytes in the vascular wall are derived from perivascular adipose tissue (PVAT); they have been closely linked with AAA progression (3). PVAT, a unique type of adipose tissue, surrounds most blood vessels (4). Recently, PVAT has attracted considerable interest in the context of vascular diseases. The fundamental regulatory role of PVAT in vascular physiology and dysfunction has been reported to affect both dilated and atherosclerotic aortic diseases (5–7). Additionally, PVAT can secrete various substances, thus promoting expansion of the AAA wall; AAA diameter in the vascular wall has been shown to significantly decrease upon removal of PVAT (8). However, the underlying mechanism by which PVAT contributes to AAAs is unclear. In this review, we focus on PVAT-derived cytokines in the pathophysiological progression of AAAs. Moreover, we highlight the potential for PVAT-derived factors to serve as a therapeutic target in the treatment of AAAs.

## Characteristics of AAA

**Figure 1:**
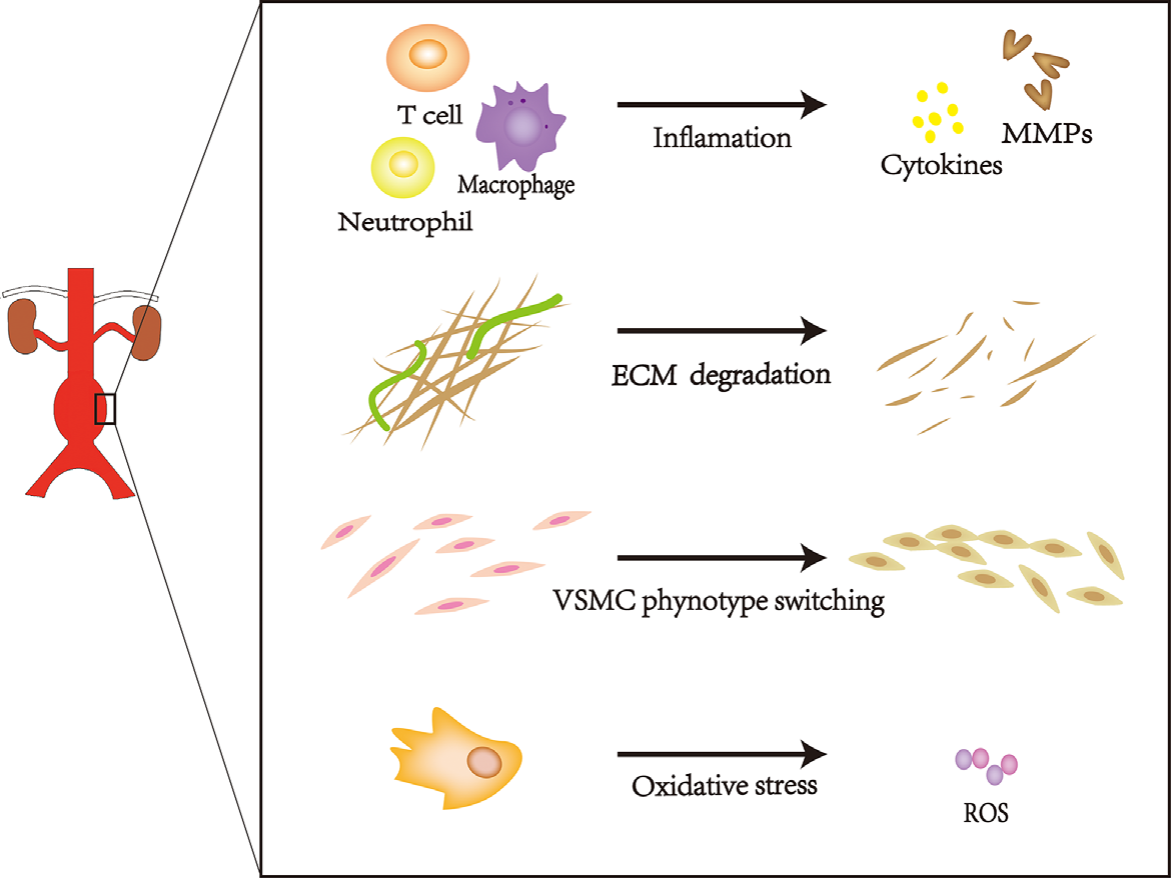
The pathogenesis of AAA.

AAAs can occur in any portion of the inferior phrenic aorta, although they frequently occur in the infrarenal abdominal aorta (9). Most AAAs are small and become increasingly prominent over time. Moreover, the risk of rupture increases with increasing aneurysm diameter (10). Age, increased smoking frequency, family history of AAAs, and a high-fat diet have been associated with AAA expansion (11); this type of change is characterized by progressive expansion and weakening of the three layers of the abdominal aorta (i.e., intima, media, and adventitia). The intima is composed of a layer of endothelial cells upon connective tissue, the media comprises vascular smooth muscle cells (VSMCs) embedded in structural proteins, and the adventitia comprises fibroblasts and collagen fibers (12). Damage to any layer of the abdominal aorta will promote AAA progression, frequently leading to rupture-related mortality. This pathological progression involves extracellular matrix (ECM) degradation, vascular smooth muscle cell phenotype switching, inflammation, and oxidative stress. (**Figure 1**) (13–16). AAAs cause more than 15,000 deaths annually in the United States; approximately 25% of patients with aortic rupture can achieve prolonged survival by undergoing surgery (16–18). Currently, AAAs cannot be treated with medication; treatment is limited to surgical repair to prevent disastrous rupture. However, this treatment does not provide substantial benefits to patients with small AAAs (19). Thus, there is a need to develop new therapies to reduce the risk of AAA rupture.

## Characteristics of PVAT

PVAT is a metabolically hyperactive tissue that surrounds many large blood vessels except the cerebral vasculature. Typical PVAT includes adipocytes, microvasculature, stromal cells, and inflammatory cells; the specific phenotype depends on anatomical location and varies markedly due to pathogenesis (20). PVAT contains both white and brown adipose tissue. White adipose tissue (WAT) stores energy in the form of triacylglycerols, which can be mobilized through lipolysis during energy expenditure or increased fasting. Additionally, WAT can secrete various hormones, cytokines, and enzymes. These substances derived from WAT are essential to biological processes such as inflammation, metabolism, and vascular homeostasis (21, 22). In contrast, brown adipose tissue (BAT) can generate heat through intracellular lipolysis and the activity of uncoupling protein 1. Intracellular lipolysis produces fatty acids as thermogenic substrates, while uncoupling protein 1 interrupts electron transport during the generation of adenosine triphosphate within the cristae-dense mitochondria in BAT (20, 23). The compositions of WAT and BAT in PVAT vary throughout the human body. PVAT contains mainly BAT in the thoracic aorta and mainly WAT in the abdominal aorta (6, 24, 25). In normal physiological conditions, PVAT releases vasoactive molecules (e.g., hydrogen peroxide, angiotensin, adiponectin, hydrogen sulfide, and nitric oxide) to attenuate agonist-induced vasoconstriction (26–28). Conversely, PVAT in pathological conditions promotes inflammation and oxidative stress; inhibits the release of vasoprotective adipocyte-derived relaxing factors; and increases the secretion of paracrine factors such as resistin, leptin, cytokines [e.g., tumor necrosis factor α and interleukin (IL)-6] and chemokines [regulated upon activation, normal T cell expressed and secreted (i.e., RANTES) and monocyte chemoattractant protein (MCP)-1] (24, 29, 30). These substances usually contribute to increased incidence of metabolic disease (e.g., obesity, diabetes, and aging), thereby promoting PVAT dysfunction and AAA progression (7, 31). Thus, PVAT has a close relationship with AAAs.

## Cellular and Molecular Contact Between Dysfunctional PVAT and AAA Pathology

**Figure 2:**
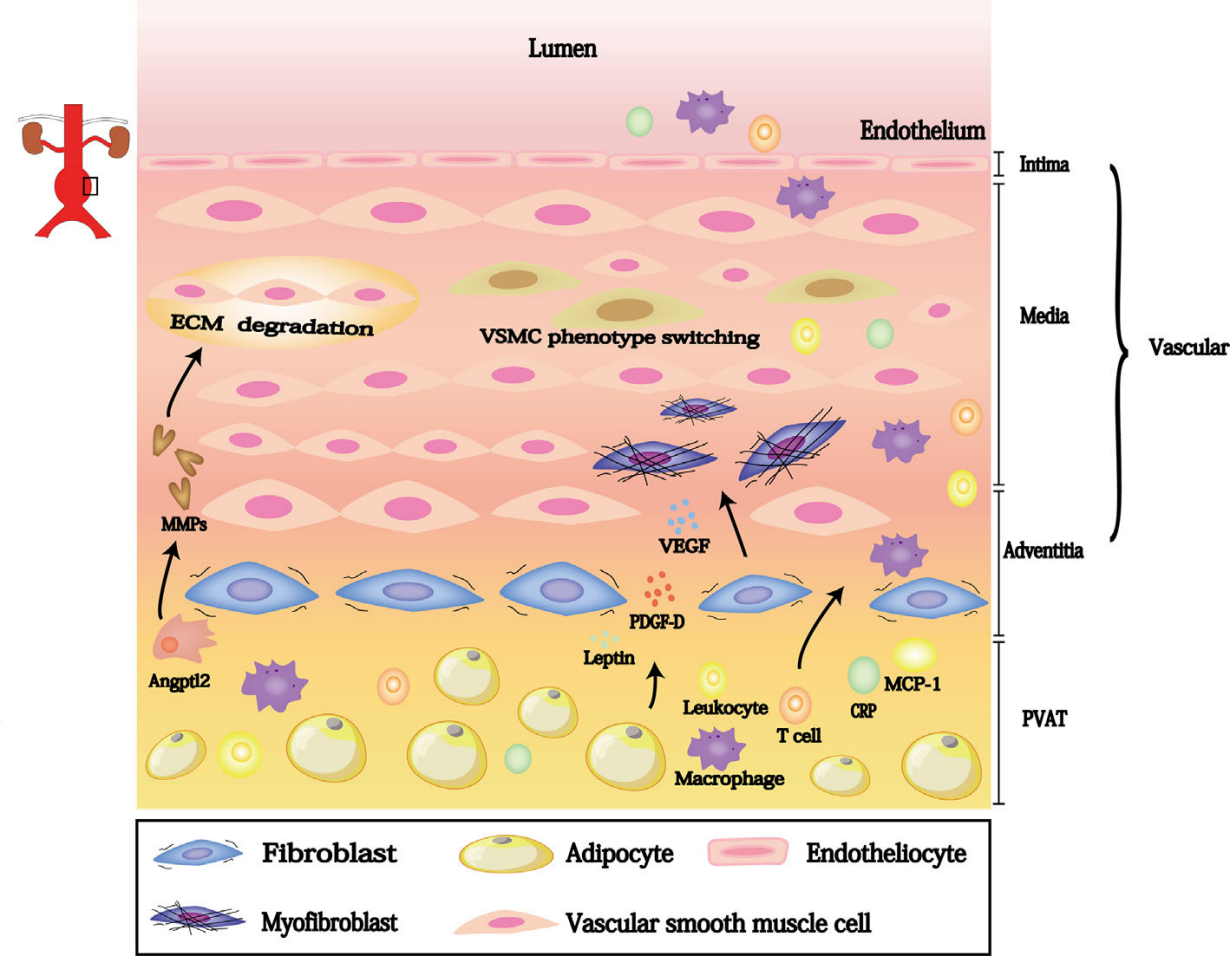
PVAT contributes to the formation of AAA under pathological conditions.

There is increasing evidence that dysfunctional PVAT influences AAA progression. This dysfunction involves inflammatory cells (e.g., lymphocyte, macrophages and neutrophil) infiltration and migration from the PVAT to the vascular wall; these cells generate reactive oxygen species to promote elastic arterial stiffness. Furthermore, PVAT can regulate neointimal formation through cytokines that promote VSMC phenotype switching, outer membrane inflammation, and neovascularization. Thus, PVAT-derived biologically active substances may participate in each stage of AAA pathogenesis; targeting these substances may aid in AAA mitigation (**Table 1** and **Figure 2**).

**Table 1:** Summary of PVAT-derived factors.

Factors	Official full name	Function in AAA	Reference
CRP	C-reactive protein	neointimal hyperplasia	(32, 33)
MCP-1	Monocyte chemoattractant protein-1	neointimal hyperplasia	(32, 33)
MMPs	matrix metalloproteinases	ECM degradation	(34, 35)
Angptl2	angiopoietin-like protein 2	neointimal hyperplasia	(36)
PDGF-D	platelet-derived growth factor-D	adventitial inflammation and fibrosis	(37)
VEGF	vascular endothelial growth factors	adventitial neovascularization	(16)
leptin		vascular remodeling	(38)
T cells	T lymphocytes	Inflammation response	(39–42)
PROK2	Prokineticin 2	inflammation and immune-related processes	(43, 44)
MAP4K1	Mitogen-activated protein kinase kinase kinase kinase 1	inflammation and immune-related processes	(43, 44)
stromal cells		vascular remodeling	(45, 46)
EVs miR-221-3p	extracellular vesicle microRNA-221-3p	VSMC phenotypic switching	(47)

### PVAT-Derived Biological Substances in AAAs

In the vascular walls of AAAs, inflammatory cytokines such as MCP-1 and C-reactive protein (CRP) are present at increased levels during pathological conditions (48). Both of these cytokines contribute to elastic arterial stiffness by promoting leukocyte and macrophage adhesion and migration into the vessel wall, leading to VSMC proliferation. These cytokines are secreted at high levels in PVAT (49), where they promote neointimal hyperplasia with macrophage infiltration and vasa vasorum proliferation after vascular damage (32, 33), thereby accelerating aneurysm formation.

Upregulation of MMPs, produced by inflammatory cells and VSMCs, are the basis of AAA pathogenesis. MMPs increase the expression levels of inflammatory substances, which disrupt critical components of ECM (e.g., elastin and collagen fibers) (50–52). ECM degradation by proteolytic enzymes, mainly MMP-2 and MMP-9, reportedly promotes AAA progression (34, 35). Notably, MMP-2 and MMP-9 expression levels are elevated by PVAT-mediated angiopoietin-like protein 2 (Angptl2) (16). Angptl2-deficient mice showed reduced AAA progression compared with wild-type mice; in particular, Angptl2-deficient mice exhibited smaller aneurysms, less vascular structural destruction, and lower MMP expression levels (53). Additionally, Tian et al. constructed wild-type mice with PVAT derived from transgenic mice expressing Angptl2 in adipose tissue; these modified wild-type mice exhibited more frequent neointimal hyperplasia after endovascular injury, compared with wild-type mice that underwent transplantation of wild-type tissue (36). These studies demonstrate that MMP inhibition in PVAT may reduce AAA size, suggesting that AAA progression can be controlled by adjusting PVAT-derived MMPs (54).

Furthermore, although PVAT-derived factors are beneficial in normal physiological conditions, long-term enhancement in pathological conditions may promote disease progression. Platelet-derived growth factor-D (PDGF-D) and vascular endothelial growth factors (VEGF) can repair damaged blood vessels, but both contribute to AAA formation. PDGF receptors are widely expressed in cells of the cardiovascular system, including fibroblasts, smooth muscle cells, and pericytes. PDGF-D signaling has important implications in fibrosis, neovascularization, atherosclerosis, and restenosis (55). A transcriptomics analysis revealed that PDGF-D was strongly expressed in PVAT from obese mice; inhibiting PDGF-D function significantly reduced AAA incidence. The experiment demonstrated that adipocyte-specific PDGF-D transgenic mice were more likely to exhibit AAA formation, accompanied by adventitial fibrosis and inflammation (37). Furthermore, Zhang et al. reported that PDGF-D stimulates the transforming growth factor-beta/small mother against decapentaplegic (i.e., Smad) pathway, thereby mediating AAA formation during obesity (37, 56). These studies showed that PVAT-derived PDGF-D has a vital role in AAAs. Importantly, PDGF-D stimulates the release of VEGF-A by fibroblasts (57). VEGF-A overexpression in PVAT facilitates adventitial neovascularization; VEGF-A is elevated in aneurysms, compared with non-aneurysmal aortae (16). The inhibition of VEGF-A expression may reduce AAA incidence (58). These findings suggested that PVAT-derived VEGF also has a vital role in AAAs. Therefore, overexpression of PVAT-derived growth factors could contribute to AAA progression by promoting adventitial inflammation.

Leptin is a robustly secreted adipokine with a secretion level directly proportionate to adipocyte size; leptin is closely involved with AAAs (59). Leptin is reportedly increased 60-fold in PVAT from obese mice, and PVAT-derived leptin was twofold greater in AAAs than in normal aortae (60). Chronic elevation of leptin could lead to vasoconstriction and VSMC phenotypic switching (61); both of these changes could accelerate exacerbate vascular remodeling and promote AAA progression (38). Additionally, PVAT-derived leptin participates in AAA pathogenesis through the IL-18 signaling pathway, which involves the IL-18 receptor and NaCl co-transporter (62, 63). Leptin can increase the expression levels of IL-18, IL-18 receptor, and NaCl co-transporter; deletions of these receptors reduced AAA growth (63). These findings suggest that PVAT-derived biological substances contribute to AAA progression.

### PVAT-Derived Immune Cells in AAAs

Immune cells from PVAT are also implicated in AAA pathogenesis. T cells are the main leukocyte subset in AAAs. Activated T cells promote the release of pro-inflammatory factors derived from macrophages in AAA models, and their greatest accumulations occur in PVAT (39). Notably, T cells are highly activated in PVAT/vascular walls, and the degree of T-cell infiltration into PVAT is strongly associated with AAA size (39–42). Furthermore, the innate immune signaling molecule CD14 has a vital role in the adventitial recruitment of macrophage precursors, which lead to AAAs; CD14 is reportedly upregulated in PVAT-conditioned medium from an AAA model *in vivo* and *in vitro* (31, 64). Thus, PVAT is a reservoir of T cells and may be critical for modulating the underlying inflammation of AAA (42).

Weighted correlation network analysis showed that prokineticin 2 (PROK2) and mitogen-activated protein kinase kinase kinase kinase 1 (MAP4K1) were hub genes in dilated PVAT samples, where they mediated AAA pathogenesis (43, 65). PROK2 is upregulated in granulocytes and macrophages within inflamed tissue; it reportedly exhibits sevenfold upregulation at AAA rupture sites (66). Furthermore, MAP4K1 expression is increased by T and B cells. Both of these proteins regulate inflammation and immune processes, such as inflammatory cell adhesion, cytokine release, and immune cell activation (43, 44). However, specific mechanisms underlying PVAT-derived gene function in AAAs remain unknown; analysis of these genes may provide promising AAA treatments.

### PVAT-Derived Stromal Cells in AAAs

Perivascular adipose tissue-derived stromal cells (PVADSCs) also participate in AAA formation (5, 67). Adipose tissue-derived stromal cells (ADSCs) are mesenchymal stem cells in essence. Cultured populations of ADSCs contain fibroblast colony-forming units and a proportion of clonable self-renewing cells, but will quit proliferating at less than 20 passages. Thus, it to be more appropriate to use ADSCs (stroma) than the term stem cells for ADSCs (68). PVADSCs can be distinguished into several cell lines under specific culture conditions, including endothelial cells, smooth muscle cells, osteoblasts, and adipocytes (69–71). This capability is particularly robust in young PVADSCs, but is weak in aged cells. Aged PVADSCs show decreased differentiation or aberrant secretion of adipokines and cytokines, which leads to reduction of their protective effects against vascular lesions. These changes could initiate myofibroblast proliferation and migration, followed by neointimal induction (45). Moreover, PVADSCs from AAA patients displayed enhanced senescence manifestation. This manifestation contains increased decreased proliferation, migration ability, mitochondrial fusion, reactive oxygen species production, and decreased mitochondrial membrane potential, which all contribute to AAA formation (46).

### PVAT-Derived Extracellular Vesicle miRNAs in AAAs

Multiple types of PVADSCs can be induced by the transfection of microRNA (miRNA) mimics (72). Based on gene set enrichment analysis, the respective expression levels of miR-27b-3p and miR-221-3p in plasma were 1.6-fold and 1.9-fold higher in patients with AAAs than in healthy controls (73). Additionally, miR-221-3p is highly expressed in obese PVAT-derived extracellular vesicles (EVs). PVAT-derived EVs containing miRNAs communicate intercellular messages in AAA pathogenesis (74, 75). Obese mice reportedly secrete large quantities of EVs containing miRNA, which induce inflammatory reactions in PVAT and VSMC phenotype switching in the abdominal aorta. In the context of obesity-associated inflammation, PVAT-derived miR-221-3p could trigger early vascular remodeling (47). Therefore, efforts to target PVAT-derived EVs could provide novel therapeutic approaches for AAAs.

## PVAT-Targeting Therapy

The only effective therapy against large AAAs or symptomatic aneurysms is open surgery or endovascular repair; however, this provides no clear benefits with respect to small AAAs. Current research regarding drugs and cells aims to identify novel effective therapeutic and preventive strategies for AAAs. Given the roles of MMPs in AAA weakening and rupture, MMPs are considered reliable targets. Some wide-spectrum MMP inhibitors have been developed as therapeutic agents for cancer; however, no trials have shown improved overall survival, and MMP inhibitors can have severe side effects (76). However, a subset of MMP inhibitors may have better effects. In particular, MMP12 is significantly increased in AAAs while peroxisome proliferator-activated receptor γ agonist could reduce MMP12 levels, thus reducing the inflammatory and oxidative statuses of PVAT (77). Furthermore, MMP-targeted imaging can be used to predict AAA progression and rupture risk. Selective MMP12 inhibitors based on 99mTc-labeled radiotracers have the potential for detecting AAA biology and predicting AAA outcome; thus, single photon emission computed tomography imaging research may be useful regarding AAAs (78).

Additionally, some experiments have been conducted to treat AAAs by VEGF or its receptor inhibition (79). VEGF-induced PVAT cell differentiation downregulates protein kinase C epsilon and p21-activated kinase 1 phosphorylation, thus negatively regulating vascular progenitor differentiation (80). Reductions of VEGF signaling-related angiogenesis have been performed to treat AAAs in mice (81). For example, anti-VEGF-A monoclonal antibody suppresses aneurysm development, while receptor tyrosine kinase inhibitor sunitinib limits AAA initiation and progression (79). These findings indicate that VEGF and its receptors have therapeutic potential.

Regenerative medicine has achieved clear therapeutic effects in various cardiovascular diseases, including AAAs. PVADSCs, as immunomodulatory cells, inhibit the activation of T lymphocytes and repolarize the phenotype of M1 macrophages to M2. PVADSCs can differentiate to functional SMC-like cells, but inhibit SMC apoptosis. In addition, PVADSCs produce essential ECM components such as collagen, elastin, and laminin. Experiments involving transplantation of cultured PVADSCs into a mouse vein graft model suggested that PVADSCs promote VSMC differentiation, thereby contributing to vascular remodeling. PVADSCs inhibits high mobility group box 1 release, leading to reductions of proinflammatory cytokines (e.g., IL-17) and protection against AAA formation (82). PVADSCs can maintain a multipotent phenotype and are easily cultured, providing a promising treatment for small AAA (68). Thus, regenerative medicine is a compelling long-term approach for preventing AAA formation. It should be noted that the targeted therapies cannot eradicate the disease, but delay its progression in the initial stages. Therefore, it can only be applied at an early stage of illness. The exact effects still need to be supported by clinical studies.

## Conclusion

In pathological conditions, PVAT becomes dysfunctional and has a vital role in AAA formation. PVAT-derived factors participate in all stages of pathological AAA formation, including inflammatory cell infiltration, oxidative stress onset, matrix metalloproteinase activation, and VSMC phenotype switching. Thus, PVAT may be a useful new target for the development of AAA therapeutic drugs. Notably, most studies thus far have used *in vitro* and *in vivo* models of AAAs. However, AAA formation in humans is a chronic process. Moreover, the mechanisms that connect PVAT-derived factors and AAAs remain unclear. Additional studies are needed to identify the mechanisms that contribute to AAA inhibition, thus alleviating the risk of AAA rupture-induced mortality and preventing AAA formation.

## Author Contributions

NH and FH: Conceptualization, Writing - Review & Editing. TY and GZ: Methodology, Software, Visualization, Writing - Original Draft. Other authors: Software. All authors contributed to the article and approved the submitted version.

## Funding

This study was supported by grants from National Natural Science Foundation of China (81870593), Natural Science Foundation of Shandong Province (ZR2020MH106), Shandong Province Higher Educational Science and Technology Program for Youth Innovation (2020KJL004).

## Conflict of Interest

The authors declare that the research was conducted in the absence of any commercial or financial relationships that could be construed as a potential conflict of interest.
